# Orthokeratinized Odontogenic Cyst: Insights From a Case Series With a Review of Literature

**DOI:** 10.1155/crid/8949890

**Published:** 2026-06-02

**Authors:** S. Leena Sankari, Mohammed A. Assiri, Sulaiman S. Alqahtani, Khadijah Mohideen, Layla Hafed, Venkatalakshmi Aparna, Bhavana Sujanamulk

**Affiliations:** ^1^ Department of Oral and Maxillofacial Pathology and Oral Microbiology, Sree Balaji Dental College and Hospital, Bharath Institute of Higher Education and Research, Chennai, Tamil Nadu, India, bharathuniv.ac.in; ^2^ Department of Oral and Maxillofacial Surgery and Diagnostic Sciences, Faculty of Dentistry, Najran University, Najran, Saudi Arabia, nu.edu.sa; ^3^ Department of Oral Medicine and Diagnostic Science, Faculty of Dentistry, Saba University, Sana′a, Yemen; ^4^ Department of Oral Medicine and Radiology, Ragas Dental College and Hospital, Chennai, Tamil Nadu, India

**Keywords:** enucleation, keratocyst, odontogenic cyst, orthokeratinized odontogenic cyst, recurrence

## Abstract

Orthokeratinized odontogenic cyst (OOC) is an uncommon developmental cyst of odontogenic origin that was considered a variant of the odontogenic keratocyst (OKC). However, extensive research has demonstrated that OOC exhibits distinctive clinical, radiological, and histopathological features, warranting its classification as a distinct pathological entity. Histologically, OOC presents a thin, uniform epithelial lining with a prominent orthokeratinized surface and a distinct granular cell layer, features that clearly differentiate it from the parakeratinized and corrugated epithelial lining typically observed in OKC. OOC shows a slow‐growing, less aggressive behavior and minimal recurrence potential. The preferred treatment is surgical enucleation, and the prognosis is excellent, with recurrence rates reported to be below 2%, in sharp contrast to the 8%–25% observed in OKC following similar management. This case series highlights six OOC cases, elaborating on their clinical, radiographic, and histopathological features to enhance clinicians′ understanding of this rare odontogenic cyst.

## 1. Introduction

Orthokeratinized odontogenic cyst (OOC) is a rare developmental odontogenic cyst that was once considered a type of odontogenic keratocyst (OKC). However, OOC has distinct histopathological features and clinical behavior that set it apart from OKC. The latter is known for its locally aggressive growth and higher chance of recurrence. OOC forms from remnants of the dental lamina or the basal cell layer of the oral mucosal epithelium [1]. Treatment for OOC typically involves enucleation, and the outlook after this procedure is excellent, with a recurrence rate of less than 2%. In contrast, OKC has a recurrence rate of 8%–25% after enucleation. Research on the immunocytochemical response of cytokeratin shows that orthokeratinized epithelium is much less aggressive. These findings highlight the importance of clearly identifying OOC and OKC to ensure proper treatment planning and accurate prognostic evaluation [2–4].

Despite its well‐recognized clinicopathologic distinction from OKC, OOC is often misdiagnosed because of its overlapping clinical and radiographic features with other odontogenic cysts and tumors. The objective of this case report is to describe a case series of six histopathologically confirmed cases of OOC and to comprehensively analyze their clinical, radiographic, and histopathological characteristics. Particular emphasis is placed on the distinguishing characteristics of OOC relative to OKC, thereby reinforcing the importance of accurate diagnosis in guiding appropriate conservative management, avoiding unnecessary aggressive treatment, and ensuring an excellent long‐term prognosis.

## 2. Case Reports

Histopathological records from the Department of Oral Pathology and Oral Microbiology of a dental teaching institution were retrospectively reviewed for cases of OOC between 2016 and 2024. A total of six cases were identified. All cases exhibited cystic lumens lined by orthokeratinized stratified squamous epithelium with a prominent granular layer and keratin flakes within the lumen. The cohort comprised three males (50%) and three females (50%), with a male‐to‐female ratio of 1:1. Patient age ranged from 13 to 45 years (mean: 30 years; median: 31 years). Five cases (83.3%) involved the mandible, whereas one case (16.7%) involved the maxilla. Clinically, four cases presented with swelling, one with pain and swelling, and one was asymptomatic and detected incidentally on a radiograph. The provisional diagnoses included odontogenic cysts and tumors such as dentigerous cyst, OKC, and ameloblastoma (Table 1).

**Table 1:** Demographic and clinical details.

Case no.	Age/gender	Site	Clinical presentation	Radiological findings	Type of biopsy	Operative findings	Provisional diagnosis
1	41/F	Left side of the mandibular ramus	Swelling	Well‐defined unilocular radiolucency in the left mandible region	Excisional	White cheesy material	Odontogenic keratocyst
2	34/F	Right side of the mandible	Pain and swelling	Well‐defined unilocular radiolucency in the right side of the mandible	Incisional	No evident cystic fluid	Ameloblastoma
3	23/M	Left side of the mandibular ramus and the retromolar region	Swelling	Well‐defined unilocular radiolucency in the left side of the mandible with impacted 38	Excisional	Pale, straw colored fluid	Dentigerous cyst
4	39/M	Left side of the mandible	Swelling	Well‐defined unilocular radiolucency in the left mandibular region with impacted 38.	Incisional	White fluid	Odontogenic keratocyst
5	27/M	Left side of the maxillary antrum	Swelling	Well‐defined unilocular radiolucency in the left side of the maxilla	Excisional	No evident cystic fluid	Ameloblastoma
6	13/F	Right side of the mandible	Incidental finding	Well‐defined radiolucency in the lower right posterior region between 46 and 47, with an adjacent unerupted third molar	Excisional	Grayish white fluid	Odontogenic keratocyst

Abbreviations: F, female; M, male.

Radiographically, all cases showed a well‐defined, unilocular radiolucency; two were associated with an impacted tooth, and one with an unerupted third molar (Figures 1, 2, and 3). The specimens included two incisional and four excisional biopsies. The excisional biopsy specimens measured 1–5 cm in the greatest dimension and were firm in consistency. The cut surfaces ranged in color from white to brownish black. Histopathological examination of hematoxylin and eosin (H&E)–stained sections revealed cystic lumens lined by orthokeratinized stratified squamous epithelium with underlying connective tissue stroma. Two cases showed thin uniform epithelium, whereas four had variable thickness. The basal layer was low cuboidal in four cases and flat in two cases; all showed a distinct granular layer. Keratin flakes were abundant in three cases, and all exhibited an onion‐skin appearance. The stroma was moderately collagenized, with diffuse inflammation in four cases and a sparse infiltrate in two cases. Vascularity was moderate in three, with hemorrhagic areas in all. Two cases showed normal bony trabeculae. All were diagnosed as OOCs, predominantly displaying thin, uneven epithelium and uniform keratin flakes, with minor variations in vascularity and inflammation (Figures 4, 5, and 6). Among the six patients, two who initially underwent incisional biopsy subsequently received complete surgical enucleation with curettage of the lesion site following histopathological diagnosis. Two patients who underwent excisional biopsy were lost to follow‐up, and therefore, no additional treatment or outcome data were available for them. The remaining four patients were followed for 6 months, during which no evidence of recurrence or other complications was observed.

**Figure 1:**
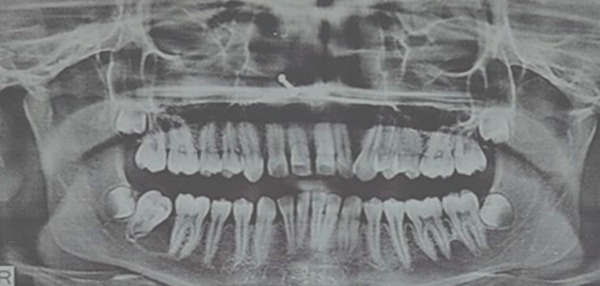
(Case 6) Orthopantomograph shows unilocular radiolucency between 46 and 47.

**Figure 2:**
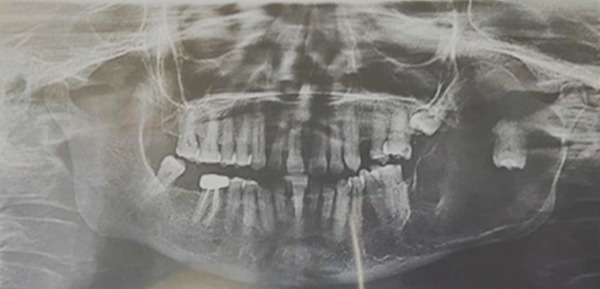
(Case 3) OPG reveals a unilocular radiolucency surrounding 36 till the ramus and subcondylar region with cortical expansion, with impacted 38.

**Figure 3:**
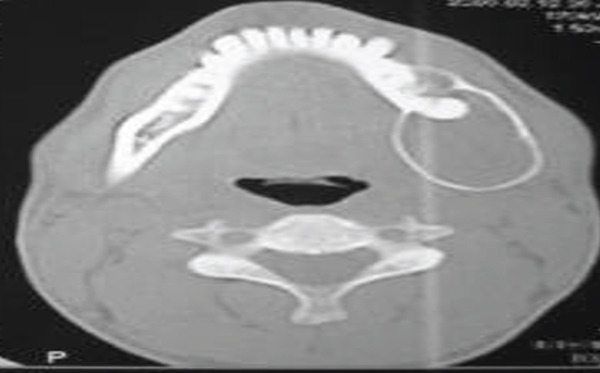
(Case 4) Axial CT section shows well‐defined unilocular pericoronal radiolucency associated with the lower right side impacted third molar tooth.

**Figure 4:**
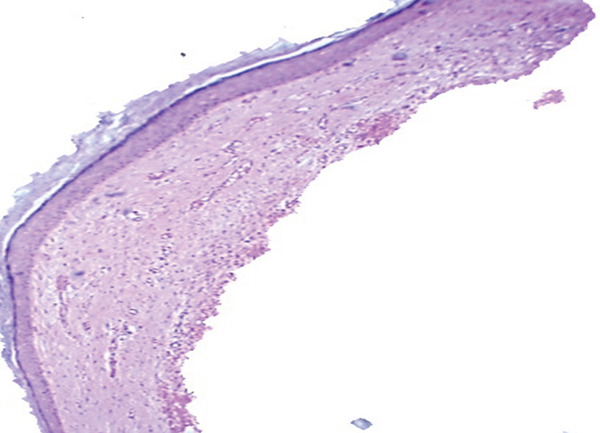
(Case 4) H&E‐stained tissue section shows orthokeratinized lining epithelium with moderately collagenized stroma in low‐power view (4×).

**Figure 5:**
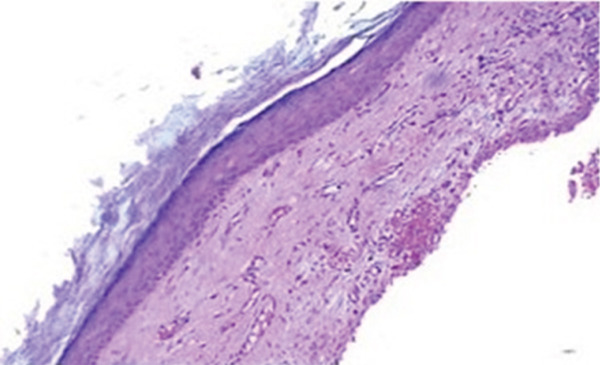
(Case 4) H&E‐stained tissue section shows orthokeratinized lining epithelium with moderately collagenized stroma in low‐power view (10×).

**Figure 6:**
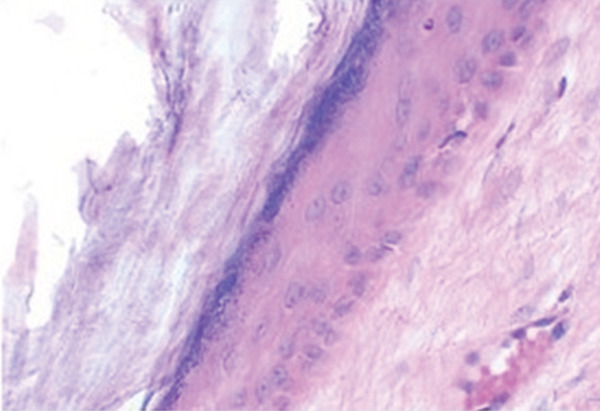
(Case 4) H&E‐stained tissue section shows orthokeratinized stratified squamous epithelium with prominent stratum granulosum in high‐power view (40×).

Written informed consent for publication was obtained from the parent or guardian of the 13‐year‐old patient, whereas direct consent was obtained from the other five patients. All identifying information has been fully anonymized to ensure patient confidentiality and privacy.

## 3. Discussion

The OOC is a type of odontogenic cyst first identified by the World Health Organization as a rare variant of the OKC [4]. This cyst makes up about 11% of all odontogenic cysts and has a female‐to‐male ratio of 2:1. It typically occurs in the mandible, particularly in the molar and ramus areas. Studies show that OOC has unique clinicopathologic features compared with other developmental odontogenic lesions, such as dentigerous cysts and OKC. Clinically, OOC may appear as a swelling that may or may not be painful and can grow significantly, causing cortical expansion. It is most often diagnosed in individuals in their third and fourth decades of life, but can also occur in younger adults, with a slight male dominance. The mandible is more frequently affected than the maxilla. Although the age of occurrence and preferred locations for OOC resemble those of OKC, the two lesions behave differently biologically, as OKC can appear in multiple locations. The size of OOC can range from less than 1 cm to larger lesions exceeding 7 cm in diameter [5].

The histogenesis of OOC is still unclear, and several theories have been proposed. OKC may arise from the dental lamina, which is influenced by the dental papilla. In contrast, OOC might come from the oral epithelium, either under the influence of the dental papilla or solely from the oral epithelium [6]. Due to the pluripotential nature of odontogenic cyst epithelium, the reduced enamel epithelium can keratinize in response to suitable stimuli. This process can lead to the formation of a true dentigerous cyst with orthokeratinization, whereas OOC may also represent a central epidermoid cyst [7, 8]. Keratin expression studies have revealed distinct keratin profiles for OOC and OKC. OOC expresses K1, K10, and Loricrin (LOR), which resembles the epidermis. Meanwhile, OKC expresses K4, K13, and K17, similar to the dental lamina. These findings suggest that OOC may arise from isolated stomodeal ectoderm during embryogenesis, supporting the idea that OOC and OKC are distinct odontogenic entities [7, 8].

They lack distinct clinical or radiographic features that set them apart from other inflammatory or developmental odontogenic cysts. Radiographically, the cyst appears as a well‐defined radiolucency that may relate to an unerupted tooth or a root without causing resorption. This radiolucency can be unilocular or multilocular and may displace adjacent teeth and the inferior dental canal. Approximately two‐thirds of OOCs are associated with impacted teeth and appear clinically and radiographically similar to dentigerous cysts [5]. The clinical and radiographic similarities with other odontogenic lesions often lead to incorrect diagnoses of OOCs. These are most frequently misdiagnosed as dentigerous cysts (44.4%), followed by inflammatory periapical lesions (25%), OKCs (19.4%), adenomatoid odontogenic tumors (8.3%), and unicystic ameloblastomas (2.7%), depending on the location and extent of the lesion [9].

Different rare forms of OKC (OOC) have been documented in the literature. These include peripheral OOC, bilateral OOC, and OOC with histopathological associations with calcifying odontogenic cyst, ameloblastoma, heterotrophic cartilage, and oral squamous cell carcinoma [10]. The main histological difference between OOC and OKC lies in the epithelial lining. The lining epithelium is thin, uniform, and orthokeratinized. The orthokeratin present on the luminal surface has an onion‐skin appearance, and the stratum granulosum is prominent. The basal layer consists of low cuboidal or flattened cells, with minimal nuclear palisading [11]. The stromal connective tissue contains collagenous fibrous tissue that may be infiltrated with chronic inflammatory cells if the cyst becomes infected [12]. A mutation in the patched homolog (PTCH) gene has been found in three cases of multiple OOC, whereas solitary OOC cases have shown no evidence of this mutation [13].

Compared with OKC, immunohistochemical findings in OOC confirm their lower aggressiveness. Ki‐67 proliferative activity and bcl‐2 antiapoptotic activity can confirm the diagnosis of OOC. Basal layer cells may display Ki‐67 positivity, whereas Bcl‐2 expression is generally absent. In contrast to benign odontogenic tumors, p63 expression is typically more intense and spread out, indicating local aggressiveness. The epithelial cells in OOC exhibit reduced proliferation and self‐renewal due to lower p63 expression. p63, a member of the p53 gene family, is crucial for the final differentiation and maintenance of epithelial stem cells. The epithelial linings in OOC exhibit fewer Ki‐67–positive proliferative cells, primarily localized to the basal cell layer, compared with OKC. The lining in OKC shows higher suprabasal proliferative activity than that in OOC. Treatment for OOC is conservative, involving enucleation or enucleation with curettage. Only 4% of OOC cases have shown recurrence [14, 15].

## 4. Conclusion

OOC is a rare, developmental odontogenic cyst that was previously considered a variant of OKC. OOC has specific histopathological features and clinical behavior. OOC is treated by enucleation, and the prognosis following enucleation is excellent, with a recurrence rate of less than 2%. On the other hand, OKC is characterized by its aggressive behavior and a relatively high recurrence rate (8%–25%), and must therefore be differentiated from OOC.

## Author Contributions

S.L.S.: concept and design, acquisition and interpretation of data; K.M., L.H., and B.S.: manuscript drafting and editing; M.A.A. and S.S.A.: critical review and final version; V.A.: acquisition and interpretation of data.

## Funding

No funding was received for this manuscript.

## Disclosure

All authors have read and approved the final version of the manuscript. Layla Hafed had full access to all data in this study and takes full responsibility for the integrity and accuracy of the data analysis.

## Consent

Patients provided written consent for the publication of their case details and accompanying images. A copy of the consent form is available upon request.

## Conflicts of Interest

The authors declare no conflicts of interest.

## Data Availability

The data supporting the findings of the case report are available in the manuscript.
